# Potential cost savings to be made by slowing cognitive decline in mild Alzheimer’s disease dementia using a model derived from the UK GERAS observational study

**DOI:** 10.1186/s12877-018-0748-9

**Published:** 2018-02-23

**Authors:** Alan Lenox-Smith, Catherine Reed, Jeremie Lebrec, Mark Belger, Roy W. Jones

**Affiliations:** 1 Eli Lilly and Company, Priestly Road, Basingstoke, RG24 9NL UK; 2 Eli Lilly and Company, Erl Wood, Windlesham, UK; 3 Eli Lilly and Company, Bad Homburg, Germany; 4 RICE (The Research Institute for the Care of Older People), Royal United Hospital, Bath, UK

**Keywords:** Alzheimer’s disease, GERAS, Cost saving, Cognition

## Abstract

**Background:**

Given the high costs associated with the care of those with Alzheimer’s disease (AD) dementia, we examined the likely impact of a reduction in the rate of cognitive decline upon cost outcomes associated with this disease.

**Methods:**

Using the group of patients with mild AD dementia from the GERAS study, generalised linear modelling (GLM) was used to explore the relationship between change in cognition as measured using the Mini-Mental State Examination (MMSE) and UK overall costs (health care and social care costs, and total societal costs) associated with AD dementia.

**Results:**

A total of 200 patients with mild AD dementia were identified. Least squares mean (LSM) ± standard error (SE) reduction in MMSE score was 3.6 ± 0.4 points over 18 months. Using GLM it was possible to calculate that this worsening in cognition was associated with an 8.7% increase in total societal costs, equating to an increase of approximately £2200 per patient over an 18-month period. If the rate of decline in cognition was reduced by 30% or 50%, the associated savings in total societal costs over 18 months would be approximately £670 and £1100, respectively, of which only £110 and £180, respectively, could be attributed to a saving of health care costs.

**Conclusion:**

This study demonstrates that there are potential savings to be made in the care of patients with AD dementia through reducing the rate of cognitive decline. A reduction in wider societal costs is likely to be the main contributor to these potential savings, and need to be further evaluated when intervention costs and cost offsets can be measured.

## Background

Dementia is a common disease associated with ageing, with 7.1% of those aged > 65 years old affected [1]. This equates to 850,000 people living with dementia in the UK in 2014 [1]. It has been estimated that 209,600 incident cases of dementia are diagnosed in people aged ≥65 years in England and Wales each year, approximately 74,000 in men and 135,000 in women [2]. Concomitant improvements in health care, however, have resulted in considerable increases in life expectancy [3], which is predicted to result in an increase in the total number of individuals with dementia in the UK to over 1 million by 2025 and over 2 million by 2051 [1].

Alzheimer’s disease (AD) is acknowledged to be the commonest cause of dementia, and is estimated to represent up to 62% of all cases of dementia in the UK [1], and 60–80% of all cases of dementia within Europe [4]. AD therefore represents the leading burden of dementia in terms of cost and resource use. The GERAS study [5] is a prospective, observational study that has assessed clinical outcomes, resource use and costs associated with AD dementia in the UK and other European countries. A previous analysis of GERAS data demonstrated that the costs associated with AD increase with increasing AD dementia severity; the total societal costs associated with AD dementia in the UK over an 18-month period were £25,865 for patients with mild AD dementia at baseline, £30,905 for moderate AD dementia and £43,560 for the moderately severe to severe group [6]. Using a different method based on Delphi panels, the Alzheimer’s Society produced similar results, where the total cost of dementia to UK society was estimated to be £26.3 billion, representing an average annual cost of £32,250 per person [1].

Given the high costs associated with the care of those with AD dementia, together with the observation that it may be possible to reduce the rate of decline of cognition in AD either by lifestyle changes [7, 8] or potentially by novel therapeutics, we examined how reducing the rate of cognitive decline could potentially influence the overall costs of care of people with AD dementia. There is currently a paucity of literature that clearly demonstrates whether a decrease in the rate of cognitive decline in patients with AD dementia is associated with any potential cost savings. As informal care costs comprise a large proportion of total societal costs associated with AD [5], reducing the rate of cognitive decline may result in cost savings due to delayed or reduced need for caregiver and social support.

Our analysis was based on modelled data derived from patients enrolled in the GERAS study within the UK, focussing on those individuals with mild AD dementia who are deemed today to experience the most marked benefits from any intervention that might ameliorate the course of the disease [9]. It was performed in order to analyse the potential size and type of savings that might be expected if the rate of decline in cognition was reduced.

## Methods

### Design

The multicentre, prospective, non-interventional GERAS observational study was conducted over 18 months in three European countries and the design has already been published [5, 6]. Briefly, patients were stratified according to disease severity at baseline: mild (Mini-Mental State Examination score [MMSE] 21–26 points), moderate (MMSE 15–20 points) or moderately severe/severe AD dementia (MMSE ≤14 points). Ethical review board approval was obtained and written informed consent provided by all participants or their caregivers. Only patients in the UK with mild AD dementia were included in the analysis reported here.

### Data collection and model details

Data were collected at each routine care visit and the Resource Utilization in Dementia (RUD) instrument [10], version RUD Complete 3.1, was used to measure healthcare resource utilisation [6]. An opportunity cost approach was used to calculate total societal costs [5, 6]. In order to explore the relationship between change in cognition (measured using MMSE score) in patients with mild AD dementia and cost outcomes (health care costs; health care and social care costs; and total societal costs) associated with AD dementia over 18 months, a *post-hoc* analysis of data from the 18-month UK GERAS database lock was performed. Missing data were handled as detailed previously [6].

Three sensitivity analyses of costs were also performed, in addition to the base-case approach, applying alternative imputation rules for missing data. Imputation of missing costs was applied separately at each cost component level (health care, social care and caregiver informal care).

Generalised linear modelling with a log link and gamma distribution was used; dependent factors included in the primary analysis were baseline cognition (MMSE) score and change in MMSE score over 18 months. The models estimated the effect of a 30% or 50% reduction in MMSE score over 18 months. The full MMSE score range is 0–30 points, with lower scores reflecting greater impairment and declining with disease progression [11], although only patients with baseline MMSE scores of 21–26 (mild AD dementia) were included in this analysis. Patients were eligible for inclusion within the primary analysis if MMSE scores had been recorded in the GERAS study at both baseline and 18 months.

### Primary analysis

Generalised linear models (GLMs), which included only baseline MMSE score and score change from baseline to 18 months, were run for each cost outcome. Only patients with baseline and 18-month data (Completers) were included in the primary analysis. The percentage changes in costs over 18 months due to decline in MMSE score were converted into actual costs using mean 18-month costs for patients with mild AD dementia of £2890 for health care costs; £10,062 for health care and social care costs; and £25,740 for total societal costs [6]. The value for total societal costs differs slightly from that previously published [6] where the costs were reported as total societal costs, whereas in the current analysis a different imputation method was applied in order to report costs for each cost component (health care, health care and social care, and total societal).

#### Sensitivity analysis 1

Sensitivity analysis 1 was run using the same covariates as the primary analysis, but based on the last observation carried forward (LOCF) for MMSE. Patients in this analysis had to have at least one post-baseline MMSE score. This analysis included just MMSE score at baseline and change in MMSE at 18 months (or LOCF).

#### Sensitivity analysis 2

A further sensitivity analysis (sensitivity analysis 2) was run where patient and caregiver baseline factors identified as being associated with costs [12] were included. Factors included in sensitivity analysis 2 were: patient age, patient gender, caregiver spouse, caregiver working for pay, and patient co-morbidities. GLMs that included baseline cognitive score and score change from baseline to 18 months, but adjusted for the aforementioned patient and caregiver factors associated with costs, were run for each cost outcome (health care; health care and social care; total societal costs) for change in MMSE score (baseline to 18 months; based on Completers and on LOCF).

## Results

A total of 200 patients with mild AD dementia from the UK GERAS study [6] were identified. Patient baseline characteristics are given in Table 1.

**Table 1:** Patient demographics at baseline for the mild AD dementia patient population (*N* = 200) in the UK GERAS study [6]

Characteristic	
Mean age in years [SD]	78.9 [6.7]
Gender, n female [%]	100 [50]
MMSE score, mean [SD]	23.1 [1.6]
Mean time since diagnosis in years [SD]	1.8 [2.1]
Caregiver is spouse, n [%]	144 [72.0]
AD medication use	
No AD medication, n [%]	31 [15.5]
Acetylcholinesterase inhibitor use, n [%]	167 [83.5]
Memantine use, n [%]	1 [0.5]
Acetylcholinesterase inhibitor + memantine, n [%]	1 [0.5]

*SD* standard deviation, *AD* Alzheimer’s disease

Mean ± standard deviation (SD) MMSE scores at baseline and 18 months were 23.1 ± 1.6 and 20.1 ± 5.3, respectively. For those 147 patients for whom data were available at 18 months, the mean ± SD change in MMSE score from baseline was a reduction of 3.2 ± 4.9 (median [interquartile range] reduction 2.0 [6.0; 0.0]); least squares mean (LSM) ± standard error (SE) reduction in MMSE score was 3.6 ± 0.4 points over 18 months (based on mixed-model repeated measures [MMRM] as described in Lenox-Smith et al. 2016) [6].

Using the GLM (primary analysis) it was possible to calculate that the observed 3.6 points reduction in MMSE was associated with an 8.7% increase in total societal costs. This is equivalent to an increase of £2239 per patient over an 18-month period (Table 2). If the rate of decline in cognition was reduced by 30% (i.e. an MMSE score decline of 2.5 points from baseline to 18 months), the associated savings in total societal costs over 18 months would be £669, of which £110 could be attributed to a saving of health care costs (Table 2). If the rate of decline in cognition was reduced by 50% (i.e. an MMSE score decline of 1.8 points from baseline to 18 months) the associated total societal cost saving over 18 months was estimated at £1133. Of this £1133 saving, £182 could be attributed to a reduction in health care costs. Thus, the greatest percentage changes in costs were observed for health care costs (8.7% and 6.2% change in costs with a 30% and 50% reduction in MMSE score change from baseline to 18 months) and health care and social care costs combined (9.2% and 6.5% for a 30% and 50% reduction, respectively), when compared with total societal costs (6.1% and 4.3% change for a 30% and 50% reduction, respectively; Table 2).

**Table 2:** Potential savings of a reduction of the rate of decline in cognition as measured by MMSE in patients in the GERAS study with mild AD dementia

MMSE score change (baseline to 18 months)	% change in costs (95% CL)	ΔCost^a^ (95% CL)	Difference in cost reduction
Health care costs			
− 3.6	12.5% (6.7%; 18.3%)	£361 (194; 529)	NA
− 2.52 (30% reduction)	8.7% (4.7%; 12.8%)	£251 (136; 370)	£110
− 1.8 (50% reduction)	6.2% (3.4%; 9.1%)	£179 (98; 263)	£182
Health care and social care costs			
− 3.6	13.1% (4.4%; 22.0%)	£1318 (443; 2214)	NA
− 2.52 (30% reduction)	9.2% (3.1%; 15.4%)	£926 (312; 1549)	£392
− 1.8 (50% reduction)	6.5% (2.2%; 11.0%)	£654 (221; 1107)	£664
Total societal costs			
− 3.6	8.7% (1.8%; 15.7%)	£2239 (463; 4041)	NA
− 2.52 (30% reduction)	6.1% (1.2%; 11.0%)	£1570 (309; 2831)	£669
− 1.8 (50% reduction)	4.3% (0.9%; 7.9%)	£1107 (232; 2033)	£1133

*MMSE* Mini-Mental State Examination, *AD* Alzheimer’s disease, *CL* confidence limits, *ΔCost* change in cost, *NA* not applicable

^a^Based on mean costs of £2890 for health care costs; £10,062 for health care and social care; and £25,740 for total societal costs

An MMSE score change of −3.6 represents the least square mean cognitive decline (based on MMSE) observed in this population over 18 months [6]. Smaller reductions in MMSE score indicate less cognitive decline

Data from sensitivity analysis 1, which examined change in MMSE based on LOCF, were similar to those derived from the primary analysis, with the observed 3.6 points reduction in MMSE associated with a 9.8% increase in total societal costs. This is equivalent to an increase of £2522 per patient over an 18-month period. If the rate of decline in cognition was reduced by 30%, the associated savings in total societal costs over 18 months would be £746, of which £110 could be attributed to a saving of health care costs. Data from sensitivity analysis 2, which examined change in MMSE adjusted for specified factors, were also similar to those from the primary analysis, with the observed 3.6 points reduction in MMSE associated with a 9.9% increase in total societal costs in the Completers analysis. This is equivalent to an increase of £2548 per patient over an 18-month period. If the rate of decline in cognition was reduced by 30%, the associated savings in total societal costs over 18 months would be £772, of which £98 could be attributed to a saving of health care costs, and a 10.8% increase in the adjusted LOCF analysis. This is equivalent to £2780 per patient over an 18-month period. If the rate of decline in cognition was reduced by 30%, the associated savings in total societal costs over 18 months would be £849, of which £104 could be attributed to a saving of health care costs.

## Discussion

The findings derived from the model presented in this analysis suggest that the potential total societal cost saving associated with reducing the decline in cognitive function as measured by MMSE in individuals with mild AD dementia by up to 50% could result in savings of up to £1133 per patient over an 18-month time period. Although these costs savings are relatively modest, and represent less than 5% of the total costs associated with the care of mild AD dementia, when multiplied by the number of mild cases of AD dementia (around 290,000 cases in the UK, based on a total dementia prevalence of 850,000 in 2015 with 55% being mild dementia and 62% attributed to AD [1]), the overall cost savings could be substantial. Moreover, it is important to consider that the majority of societal costs associated with care for community-dwelling individuals with AD dementia are due to informal care [5, 6]. Although in absolute terms, the highest contribution to the potential cost savings is a reduction in caregiver time, a proportionally greater reduction in costs could be observed for health care costs and health care and social care costs combined. These findings illustrate the importance of understanding the full societal burden of AD dementia, particularly given that costs associated with the use of new treatments would be met by the health care sector, while cost offsets would be predominantly within other sectors. This emphasises the importance of exploring alternative ways of valuing and funding new interventions in AD such as having unified social and health care budgets as exemplified in the scheme currently being piloted in Manchester [13].

There has been a paucity of studies modelling the relationship between AD dementia progression and costs, and the potential savings that may be made by slowing the rate of this decline. A previous study by Wolstenholme and colleagues [14] that modelled the relationship between disease progression and cost of care in dementia noted that each one-point decline in the MMSE score was associated with a £56 increase in four-monthly costs, whereas each one-point fall in the Barthel index (a measure of functional ability) was associated with a £586 increase in four-monthly costs [14]. Other modelling studies have sought to assess the cost-effectiveness of the symptomatic treatment of AD dementia, with acetylcholinesterase inhibitors (AChEIs) initially not being considered a cost-effective use of NHS resources in mild to moderately severe AD dementia [15], however elsewhere donepezil was considered to be highly cost effective in the treatment of patients with mild-to-moderately severe AD dementia [16] and it has been suggested that galantamine may be cost effective in those with mild to moderately severe AD dementia [17]. Such disparities could be attributed to methodological differences between these modelling studies, and NICE did later recommend the use of AChEIs in mild-to-moderate AD dementia [18].

There is increasing evidence that it is possible to alter the progression of cognitive decline via lifestyle changes in patients with, or who are at risk of, dementia which may or may not be due to Alzheimer’s disease [7, 19, 20]. In addition, clinical studies of disease modifying agents for the treatment of AD dementia have attempted to reduce the decline in cognitive function associated with AD dementia [9, 21–24]. The outcomes of these studies have been mainly negative, although many studies are still ongoing.

Our previous analysis of resource utilisation, costs and clinical outcomes in non-institutionalised patients with AD dementia in the UK revealed that the mean cost associated with mild AD dementia over an 18-month period was £25,865, compared with £30,905 for those with moderate disease [6]. This analysis revealed that, over an 18-month time period, 44% of patients diagnosed with mild AD dementia at baseline remained in the mild state at 18 months, with only 37% progressing to moderate severity, and a further 12% becoming moderately severe/severe. It could be anticipated that costs associated with AD may be lower for patients with mild AD dementia at baseline who experienced less progression during the 18 months of the GERAS study. However, at present there is no clear understanding of the potential clinical significance of a relative decline in MMSE score (e.g. a 2.5- vs 3.6-point decline) and how this may impact on functional ability. Assuming the association between decreased cognitive decline and cost reduction observed in our study generalises to a situation where a reduction in cognitive decline is obtained via an intervention, potential cost savings/offsets are likely to occur if patients diagnosed early with mild AD dementia could be successfully treated to reduce the rate of progression; however, any costs associated with the intervention will also need to be considered.

Key strengths of this study include its prospective nature, and that it is based on real-world data derived from community-dwelling patients with AD dementia within the UK. In addition, we can have confidence that these data are robust as the estimated costs are similar to those published by the Alzheimer’s Society. There is currently no established method for the assessment of potential cost savings resulting from a reduced rate of cognitive decline, and our approach could be used in further research. It is hoped that this analysis will prompt further attempts to fully elucidate any potential cost savings.

One limitation of our analysis is that the inferred relationship between decreased cognitive decline and cost reduction is based on observational association. It is reasonable to expect that such a relationship generalises to situations where a reduction in cognitive decline is obtained via an intervention (whether pharmacological or not); however, this cannot be guaranteed. In addition, the patients enrolled in the GERAS study are community based, and not necessarily representative of all AD dementia patients, due to the requirement for patients to have a caregiver who was willing to participate in the study, and the enrolment of patients from specialist secondary care clinics. It should also be noted that not all patients demonstrated a decline in cognitive function during the course of the study, which may be for a variety of reasons including the variable natural history of the disease and that the diagnosis of AD may not always have been correct. However, although these patients probably incurred lower costs, costs associated with their care were still attributed to AD dementia. Moreover, in this analysis we have focussed on individuals with mild AD dementia only, who form the population currently being targeted for treatment in current clinical trials, most probably due to the assumption that they will be best positioned to continue to lead a relatively normal life if their rate of cognitive decline is slowed; as such, the question of potential cost savings in those with moderate or more severe disease is not addressed here but is an obvious focus for future research. Lastly, this study is only 18 months long and it is likely that over a longer period greater savings might be expected.

## Conclusions

This study demonstrates that there are potential savings to be made in the care of patients with AD dementia by reducing the decline in cognition in those patients who present with mild AD dementia. Most of the potential savings will likely occur due to a reduction in societal costs rather than those spent on health care. It is therefore important that all costs are considered when assessing the cost effectiveness of future therapies for AD. Such an approach would be helped by exploring alternatives for valuing and funding new interventions in AD. Further studies are warranted to look at the longer-term benefits of reducing the decline in cognition in patients with mild dementia due to AD.

## Abbreviations

AChEIs: Acetylcholinesterase inhibitor
AD: Alzheimer’s disease
MMSE: Mini-Mental State Examination
NICE: National Institute of Care and Health Excellence
RUD: Resource Utilization in Dementia
